# MicroRNA-7a/b Protects against Cardiac Myocyte Injury in Ischemia/Reperfusion by Targeting Poly(ADP-Ribose) Polymerase

**DOI:** 10.1371/journal.pone.0090096

**Published:** 2014-03-03

**Authors:** Bin Li, Rui Li, Chun Zhang, Hong-jun Bian, Fu Wang, Jie Xiao, Shan-wen Liu, Wei Yi, Ming-xiang Zhang, Shuang-xi Wang, Yun Zhang, Guo-hai Su, Xiao-ping Ji

**Affiliations:** 1 Key Laboratory of Cardiovascular Remodeling and Function Research, Chinese Ministry of Education and Chinese Ministry of Health, Department of Cardiology, Qilu Hospital, Shandong University, Jinan, Shandong, People's Republic of China; 2 Department of Health care, Jinan Central Hospital affiliated to Shandong University, Jinan, Shandong, China; 3 Department of Cardiology, Laizhou People's Hospital, Laizhou, Shandong, China; 4 Department of Emergency, Shandong Provincial Hospital affiliated to Shandong University, Jinan, Shandong, China; 5 Department of Emergency, Linyi People's Hospital, Linyi, Shandong, China; 6 School of Mechanical Engineering, Shandong University, Jinan, Shandong, China; 7 Engineering Training Center, Shandong University, Jinan, Shandong, China; University Heart Centre Freiburg, Germany

## Abstract

**Objectives:**

MicroRNA-7 (miR-7) is highly connected to cancerous cell proliferation and metastasis. It is also involved in myocardial ischemia-reperfusion (I/R) injury and is upregulated in cardiomyocyte under simulated I/R (SI/R). We aimed to investigate the role of miR-7 during myocardial I/R injury *in vitro* and *in vivo* and a possible gene target.

**Methods and Results:**

Real-time PCR revealed that miR-7a/b expression was upregulated in H9c2 cells after SI/R. Flow cytometry showed SI/R-induced cell apoptosis was decreased with miR-7a/b mimic transfection but increased with miR-7a/b inhibitor in H9c2 cells. In a rat cardiac I/R injury model, infarct size determination and TUNEL assay revealed that miR-7a/b mimic decreased but miR-7a/b inhibitor increased cardiac infarct size and cardiomyocyte apoptosis as compared with controls. We previously identified an important gene connected with cell apoptosis -- poly(ADP-ribose) polymerase (PARP) -- as a candidate target for miR-7a/b and verified the target by luciferase reporter activity assay and western blot analysis.

**Conclusions:**

miR-7a/b is sensitive to I/R injury and protects myocardial cells against I/R-induced apoptosis by negatively regulating PARP expression *in vivo* and *in vitro*. miR-7a/b may provide a new therapeutic approach for treatment of myocardial I/R injury. Poly(ADP-ribose) polymerase.

## Introduction

MicroRNAs (miRNAs) are single-stranded, small noncoding RNAs [1]–[3] that negatively regulate gene expression via degradation or translational inhibition of their target mRNAs [4]–[7] at the post-transcriptional level [8]–[10]. miRNAs play an important role in myocardial ischemia-reperfusion (I/R) injury [11]–[17]. For example, ectopic expression of miR-144 and -451 augmented cardiomyocyte survival, which was further improved by overexpression of miR-144/451 in response to simulated myocardial I/R injury [12]. Knockdown of endogenous miR-320 protects against I/R-induced cardiomyocyte death and apoptosis by targeting heat shock protein 20 (Hsp20) [13]. miR-494 targets both proapoptotic and antiapoptotic proteins for cardioprotective effects against I/R-induced injury by activating the Akt pathway [15].

As the most abundant homologue of the poly(ADP-ribose) polymerase (PARP) family [18], PARP-1 is an affluent nuclear enzyme and plays a pivotal role in DNA repair, regulation of cytoskeletal organization, expression of various proteins and genes, and apoptosis [19]. As the substrate of caspase-3, the 116-kDa nuclear PARP is cleaved into an 89-kDa apoptotic fragment during cell apoptosis [20]; The activation of caspase-3 and proteolytic cleavage of PARP are considered major executioners of apoptosis [21]. Heart I/R injury triggers apoptotic cell death by activating a caspase cascade and cleavage of PARP, and inhibition of PARP can promote cell and tissue survival and reduce cell apoptosis.

miR-7, as a tumor suppressor, reduces proliferation and increases apoptosis of tumour cells such as in malignant neuroblastomas, lung cancer and tongue squamous cell carcinoma [22]–[26]. However, the role of miR-7a/b in cardiac I/R injury has rarely been reported. Microarray assay revealed that miR-7 was upregulated in murine hearts after I/R [13]. In this study, we detected the expression of miR-7a/b with real-time PCR and investigated its role during myocardial I/R injury *in vivo* and investigated its role during myocardial I/R injury *in vivo* and a possible target.

## Methods

### Cell culture

H9c2 (rat ventricular cell line) and HEK293 (human embryonic kidney cell line) cells were obtained from the American Type Culture Collection (Manassas, VA). The cells were cultured at 37°C under 5% CO_2_ in Dulbecco modified Eagle medium (DMEM) containing 10% fetal bovine serum (Invitrogen-Gibco) and 100 µg/ml penicillin/streptomycin.

### Simulated ischemia/reperfusion (SI/R)

At 48 h after transfection with miR-7a/b mimic or inhibitor (see below), H9c2 cells were subjected to SI/R. Specifically, the medium was replaced by serum- and glucose-deficient DMEM [27], and cells were placed into a hypoxic chamber at 37°C for 10 h, then were reoxygenated for 2 h with DMEM containing 10% fetal bovine serum.

### Cardiac I/R animal model

The animal experiments conformed to the Animal Management Rules of the Chinese Ministry of Health (document No. 55, 2001) and were approved by the Animal Care Committee of Shandong University. Female Wistar rats (12–16 weeks old, from Shandong University) were housed in an animal holding facility under standard light (alternating 12 h light/dark cycles), temperature (22°C±0.5°C) and humidity (60%±10%) for at least 1 week before experiments. Rats were randomly divided into 7 groups for treatment (n = 16 each): 1) sham control; 2) I/R: the left anterior descending branch (LAD) was occluded for 30 min and reperfused for 2 h; 3) I/R+GFP: I/R after injection of lentivirus vector with a GFP reporter into the rat myocardium [28] as a negative control for 7 days; 4,5) I/R+miR-7a/b mimic: IR after injection of lentivirus vector with miR-7a/b mimic into the rat myocardium for 7 days; 6,7) I/R+miR-7a/b inhibitor: I/R after injection of lentivirus vector with miR-7a/b inhibitor into the rat myocardium for 7 days.

For the I/R procedure, rats were anesthetized with sodium pentobarbital (50 mg/kg intraperitoneally). The trachea was cannulated with a PE-90 catheter, and artificial respiration was provided by a respirator with fraction of inspired oxygen (FiO_2_) 0.80, frequency 100 strokes/min and tidal volume 0.8 to 1.2 mL to maintain normal partial pressure of O_2_, partial pressure of CO_2_ and pH. The heart was exposed by left thoracotomy in the fourth intercostal space. The I/R model was induced with a 4–0 silk suture ligating LAD to block blood flow. After 30 min of ischemia, the knot was relaxed and the heart was allowed to reperfuse for 2 h. Rats were then killed. Sham control animals underwent the entire surgical procedure and the silk suture was passed beneath the coronary artery, but the LAD was not ligated.

### Infarct size determination

At the end of reperfusion, the rat LAD coronary artery was re-occluded and Evans blue dye solution (3 mL, 2% wt/vol) was injected into the left ventricle to identify ischemia (area at risk [AAR]) and non-ischemia (area not at risk). Then hearts were harvested and rinsed in normal saline. Tissues were semi-frozen for 30 min at −20°C, then the left ventricle was isolated and transversely cut into slices (1 mm thick). Slices were perfused with 1% triphenyltetrazolium chloride (Sigma) at 37°C for 15 min to distinguish ischemic and infarcted tissue. Non-infarcted areas with blue staining were designated as viable, and infarcted areas without staining were designated as non-viable. Finally, after the areas of the ventricle were weighed separately, the AAR and infarct size were calculated, and the infarct size was expressed as a percentage of the AAR [29].

### MiRNA transfection

The miR-7a/b mimic sequences were designed as follows: 5′-UGGAAGACUAGUGAUUUUGUUGU-3′/5′- UGGAAGACUUGUGAUUUUGUUGU-3′. The miR-7a/b inhibitor sequences were designed as follows: 5′-ACAACAAAAUCACUAGUCUUCCA-3′/5′- ACAACAAAAUCACAAGUCUUCCA-3′. The scramble control miRNA was synthesized with the following sequence: 5′-UUCUCCGAACGUGUCACGUTT-3′. The anti-microRNA control sequence was 5′-CAGUACUUUUGUGUAGUACAA-3′. All sequences were obtained from Genepharma (Shanghai). H9c2 cells were seeded at 1×10^6^ per 100-mm plate overnight until 50% confluence. For 1×10^6^ cells, 0.4 nmol miR-7a/b mimic or inhibitor was mixed with 5.0 µl Lipofectamine 2000 (Invitrogen, USA) and transfected into cells following the manufacturer's instructions. After 6 h, the supernatant was removed and fresh medium was added. Cells were cultured for another 48 h before further experiments [30].

### Quantitative RT-PCR

Total RNA including miRNA was isolated with TRIzol reagent (Invitrogen) according to the manufacturer's instructions. cDNA synthesis involved use of the TaqMan MicroRNA Reverse Transcription Kit (Applied Biosystems) with RT-U6 and miRNA-specific stem-loop primers. miR-7a/b levels were measured by use of Taqman MicroRNA assays (Applied Biosystems) and Taqman Universal PCR Master Mix (Applied Biosystems). For PARP mRNA quantification, cDNA synthesis was performed with Oligo(dT). Quantitative RT-PCR analysis was performed with a SYBR RT-PCR kit (Takara). The comparative Ct (threshold cycle) value was used for measurement with U6 and β-actin as an internal control.

### Western blot analysis

Equal amounts of protein were isolated from cultured H9c2 cells and western blot analysis was performed with the anti-PARP antibody (dilution 1∶1000; 116 and 89 kDa) and anti-caspase-3 antibody (1∶1000; 35 and 17 kDa; both Cell Signaling Technology, USA). Immunoreactive bands were visualized by use of an enhanced chemoluminescence kit (Millipore, USA) and quantified by use of Image-Pro Plus 6.0.

### Flow cytometry

After SI/R, H9c2 cells were collected and resuspended in 1× binding buffer, then these cells were stained with antibody to Annexin V-FITC and propiduim iodide (BD Biosciences, Franklin Lakes, NJ) according to the manufacturer's instructions. The percentage of apoptotic cells was quantified by flow cytometry (BD, USA).

### Luciferase assay

After miR-7a/b mimic was transfected into HEK293 cells, GV126-PARP-3′-UTR-WT (wild type)/MU (mutant type) vectors expressing firefly luciferase and pRL-cmv vectors expressing renilla luciferase (Genechem,Shanghai) were cotransfected into cells by use of Lipofectamine 2000 (Invitrogen). Luciferase activity was measured with the Dual-Luciferase Reporter Assay System (Promega) and renilla luciferase activity was an internal control.

### Terminal deoxynucleotidyltransferase-mediated dUTP nick end labelling (TUNEL)

We used TUNEL assay to evaluate apoptotic activity by DNA breakage. Rat heart sections were deparaffinized and rehydrated with serial changes of xylene and ethanol, then preprocessed with freshly prepared permeabilisation solution containing 0.1% Triton X–100 and 0.1% sodium citrate for 15 min for optimal proteolysis. Apoptosis was detected with an apoptosis detection kit (Roche, Germany). The TdT reaction was carried out for 1 h at 37°C in a humidified chamber, and sections were stained with DAPI for nuclei. TUNEL-positive cells were counted in a random selection of 10 fields and expressed as a percentage of normal nuclei [31] under confocal laser microscopy.

### Lactate dehydrogenase (LDH) release assay

Before rats were killed, serum was collected and LDH concentrations were measured with a commercial kit (Jiancheng, China).

### Statistical analysis

All data are expressed as mean ±SD. Student's *t* test and ANOVA were used for statistical evaluation. P<0.05 was considered statistically significant.

## Results

### miR-7a/b expression upregulated by SI/R in H9c2 cells

**Figure 1:**
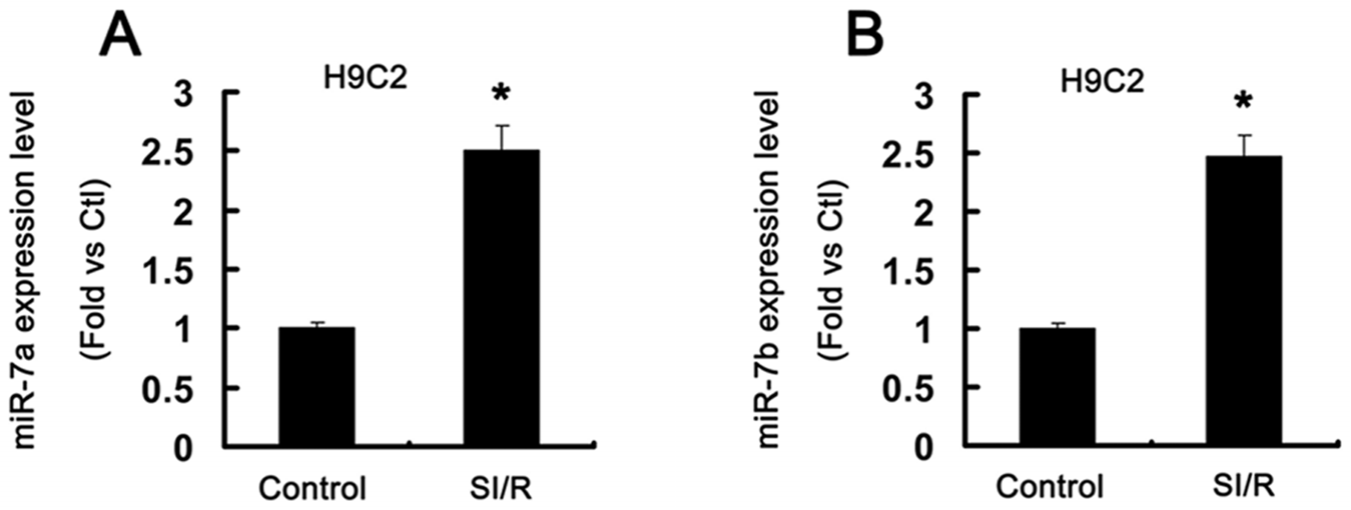
miR-7a/b expression was upregulated in H9c2 cells after simulated ischemia-reperfusion (SI/R). H9c2 cells underwent hypoxia for 10(A) Expression of miR-7a. (B) Expression of miR-7b. N = 9, *P<0.01 vs. control.

To identify the potential effect of miR-7a/b in myocardial I/R injury, we measured the expression of miR-7a/b in H9c2 cells after 10 h hypoxia and 2 h reoxygenation (SI/R). As compared with controls, the level of miR-7a/b was increased by 2.5- and 2.46-fold, respectively, after SI/R (P<0.01, Fig. 1A,B). Therefore, miR-7a/b may play an important role in SI/R of H9c2 cells.

### miR-7a/b directly targeted PARP and downregulated PARP protein expression in H9c2 cells

**Figure 2:**
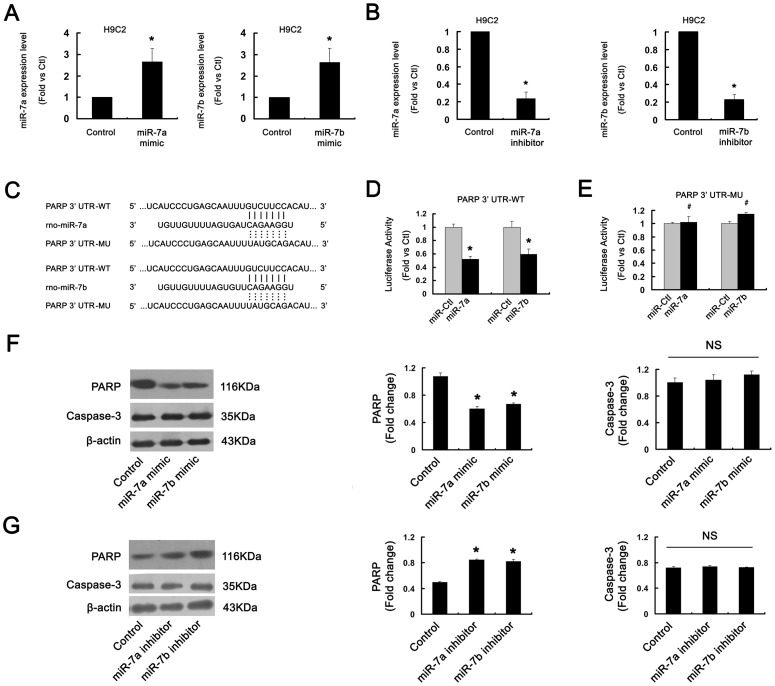
miR-7a/b targets poly(ADP-ribose) polymerase (PARP). (A,B) qRT-PCR of the efficiency of transfection of miR-7a/b mimic or inhibitor. N = 9, *P<0.01 vs. the control. (C) Conserved miR-7a/b binding site in 3′ untranslated region (UTR) of PARP. WT: wild type; MU: mutant type. (D, E) After miR-7a/b mimic or negative control miRNA was transfected into HEK293 cells, luciferase reporters were cotransfected into HEK293 cells for 48 h. Luciferase activity was measured and was normalized to that of Renilla. N = 6, *P<0.01, #P>0.05 vs. control. (F, G) Western blot analysis of protein level of PARP and caspase-3 with miR-7a/b mimic or inhibitor transfected into H9c2 cells. N = 3, *P<0.01 vs. control. NS, not significant.

To illuminate the function of miR-7a/b in H9c2 cells, we synthesized the sequences of miR-7a/b mimic and inhibitor and detected their efficiency after tansfection. The expression of miR-7a/b was greatly increased after transfection with miR-7a/b mimic, but decreased after transfection with miR-7a/b inhibitor for 48 h in H9c2 cells as compared with controls (P<0.01, Fig. 2A, B).

To elucidate the molecular mechanisms of miR-7a/b in SI/R injury of cardiomyocyte, we used TargetScan, the miRNA target prediction program (http://www.targetscan.org/), to screen putative targets and found that PARP had conserved binding sites for miR-7a/b (Fig. 2C). To confirm whether miR-7a/b targeted PARP through its 3′ untranslated region (UTR), the 3′ UTR segment of PARP mRNA harboring the putative miR-7a/b binding sequence was cloned into a firefly luciferase reporter plasmid (GV126-PARP 3′-UTR-WT), and a mutant luciferase reporter vector was constructed with the miR-7a/b binding site mutated (GV126-PARP 3′-UTR-MU) (Fig. 2C). After miR-7a/b mimic or negative control miRNA transfection into HEK293 cells, luciferase reporters were cotransfected for 48 h. Transfection with the miR-7a/b mimic strongly inhibited luciferase activity of the GV126-PARP 3′-UTR-WT (P<0.01, Fig. 2D), but not the GV126-PARP 3′-UTR-MU (P>0.05, Fig. 2E). Therefore, miR-7a/b directly targeted PARP.

To further verify whether miR-7a/b targets PARP under physiological conditions and inhibits endogenous PARP expression, we transfected miR-7a/b mimic or inhibitor into H9c2 cells, and measured the protein expression of PARP. The expression of PARP was downregulated with miR-7a/b mimic (P<0.01, Fig. 2F) and upregulated with miR-7a/b inhibitor (P<0.01, Fig. 2G). However, the expression of caspase-3 was not changed with miR-7a/b mimic or inhibitor transfection(P>0.05, Fig. 2F, G). Thus, miR-7a/b may directly target and inhibit PARP protein expression in H9c2 cells.

### Overexpression of miR-7a/b protected H9c2 cells and reduced SI/R-induced cell apoptosis

**Figure 3:**
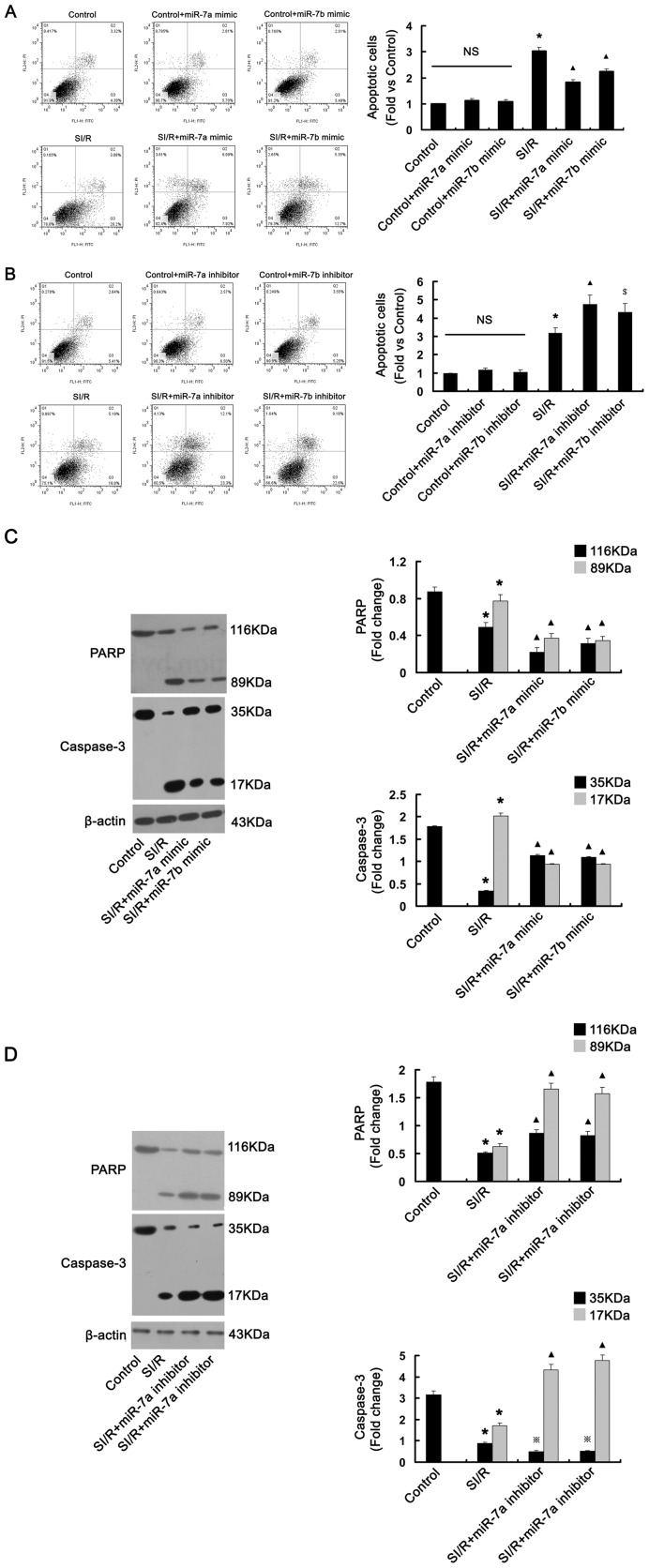
Effect of miR-7a/b on SI/R-induced cardiomyocyte apoptosis. (A, B) Cells were stained with antibody to Annexin V-FITC and propiduim iodide after transfection with miR-7a/b mimic or inhibitor; Representative flow cytometry of apoptosis of cardiomyocyte under different conditions. N = 9, *P<0.01 vs. control; ▴P<0.01, $P<0.05 vs. SI/R. NS, not significant. (C, D) Western blot analysis of the protein level of intact and cleaved fragments of PARP and caspase-3. Before SI/R, cardiomyocyte were transfected with miR-7a/b mimic or inhibitor, respectively. N = 3, *P<0.01 vs. control; ▴P<0.01, 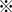
P<0.05 vs. SI/R.

Because miR-7a/b expression was upregulated by SI/R in H9c2 cells, we wondered whether miR-7a/b protected cardiomyocyte against SI/R-induced cell apoptosis. Flow cytometry revealed greater apoptosis with SI/R than control treatment in H9c2 cells. (P<0.01, Fig. 3A, B). Transfection with miR-7a/b mimic decreased the apoptosis rate (P<0.01, Fig. 3A) and Transfection with miR-7a/b inhibitor increased the rate as compared with SI/R alone (P<0.01 and P<0.05, Fig. 3B). Thus, overexpression of miR-7a/b protected H9c2 cells against SI/R-induced apoptosis.

To further investigate the mechanism of miR-7a/b protecting against cardiomyocyte apoptosis in SI/R, we transfected miR-7a/b mimic or inhibitor into H9c2 cells and evaluated the protein level of PARP and caspase-3. After SI/R, the level of intact 35-kDa caspase-3 was reduced and that of the 17-kDa cleaved fragment was increased (P<0.01, Fig. 3C, D). As the substrate of caspase-3, the 116-kDa PARP was cleaved into an 89-kDa fragment (P<0.01, Fig. 3C, D). The increased levels of cleaved PARP and caspase-3 are a hallmark of apoptosis. Under SI/R, transfection with miR-7a/b mimic significantly inhibited the protein expression of 116-kDa PARP and reduced the levels of cleaved PARP and caspase-3 as compared with SI/R alone (P<0.01 for both, Fig. 3C). In contrast, miR-7a/b inhibitor increased the protein expression of 116-kDa PARP and augmented the levels of cleaved PARP and caspase-3 as compared with SI/R alone (P<0.01 for both, Fig. 3D). Therefore, PARP is a functional gene target involved in miR-7a/b–mediated cardiomyocyte protection against SI/R-induced cell apoptosis.

### miR-7a/b inhibited PARP expression and decreased cardiac infarct size and myocardial cell apoptosis after I/R *in vivo*

**Figure 4:**
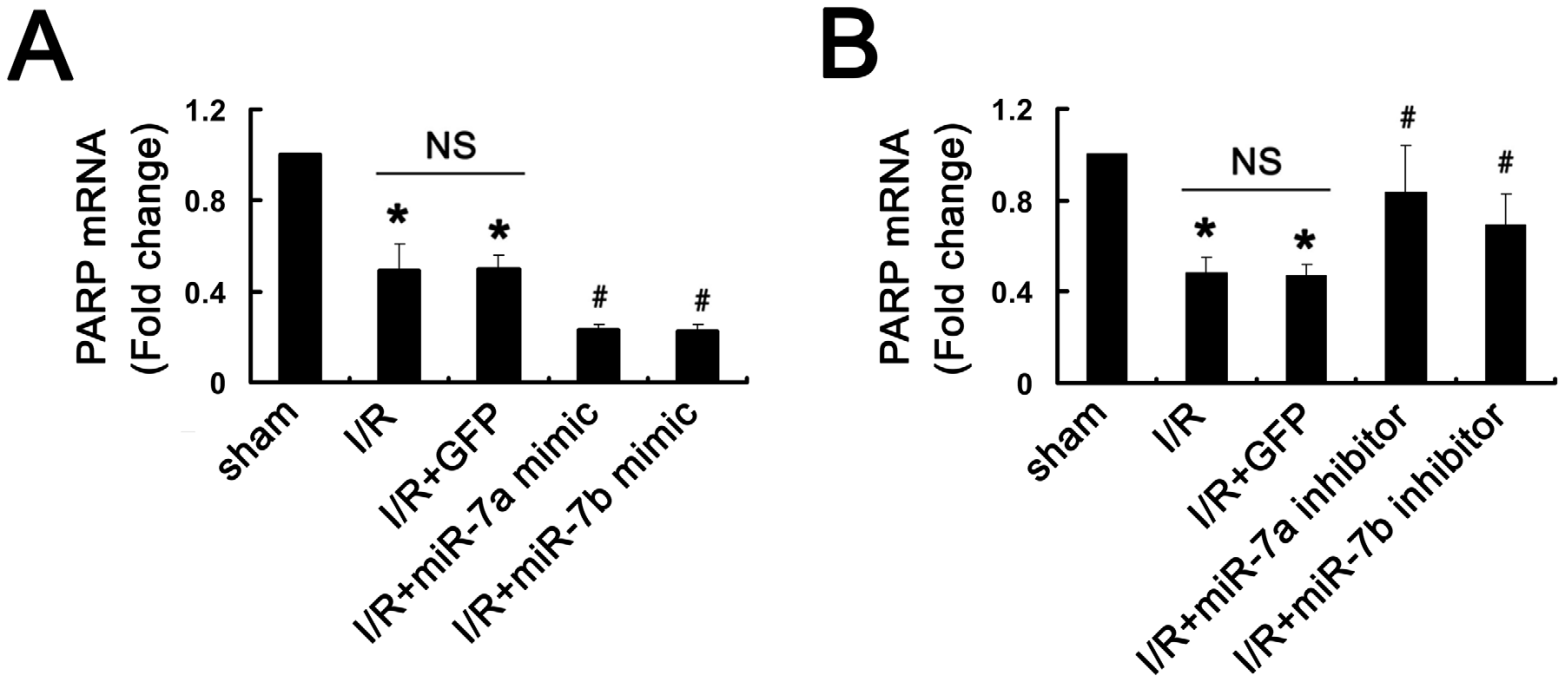
PARP is a target of miR-7a/b in the rat hearts. qRT-PCR analysis of the mRNA expression of PARP after transfection of (A) miR-7a/b mimic and (B) miR-7a/b inhibitor during I/R. N = 8, *P<0.01 vs. sham; #P<0.01 vs. I/R+GFP. NS, not significant.

We showed that miR-7a/b targets PARP *in vitro*, so we investigated whether miR-7a/b targeted PARP and inhibited PARP expression *in vivo*. The lentivirus with miR-7a/b mimic or inhibitor was injected into the rat myocardium. After successful induction of I/R in rats, the expression of PARP was decreased as compared with sham controls (P<0.01, Fig. 4A, B). PARP was downregulated with miR-7a/b mimic and upregulated with miR-7a/b inhibitor as compared with I/R+GFP (P<0.01, Fig. 4A, B). Thus, miR-7a/b may directly target and inhibit PARP expression in rat hearts.

**Figure 5:**
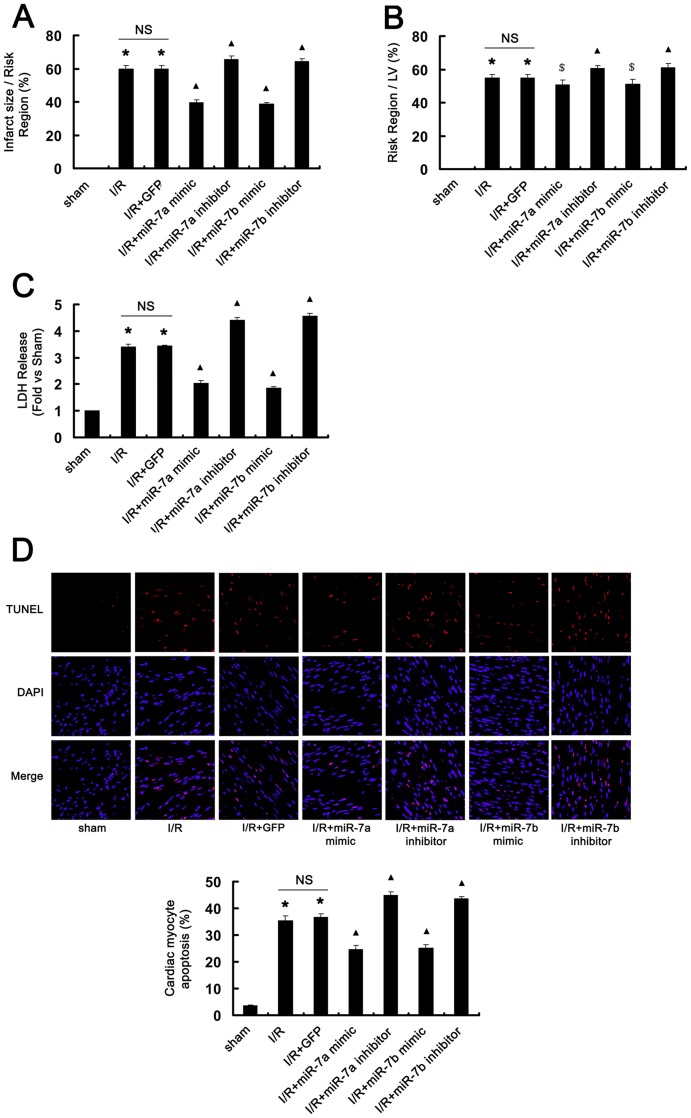
Effect of miR-7a/b on myocardial I/R injury. (A, B, C) Effect of miR-7a/b mimic and inhibitor on I/R-induced (A) infarct size, (B) area at risk, and (C) lactate dehydrogenase (LDH) release. LV, left ventricle. N = 8. *P<0.01 vs. sham; ▴P<0.01, $P<0.05 vs. I/R+GFP. (D) Representative photomicrographs and quantification of ventricular tissue with TUNEL staining under different conditions with miR-7a/b mimic or inhibitor treatment. N = 8. *P<0.01 vs. sham; ▴P<0.01 vs. I/R+GFP. DAPI, nuclei staining. NS, not significant.

To further study the biological role of miR-7a/b after I/R, we evaluated the extent of AAR and myocardial infarct size in the rat I/R injury model. Overexpression of miR-7a/b significantly reduced the infarct size (P<0.01, Fig. 5A) and AAR (P<0.05, Fig. 5B) as compared with I/R+GFP. Conversely, inhibition of miR-7a/b increased the infarct size and AAR as compared with I/R+GFP (P<0.01, Fig. 5A, B). Additionally, as a biochemical marker of myocardial cell necrosis, after I/R, LDH release was significantly suppressed with miR-7a/b mimic treatment but increased with miR-7a/b inhibitor treatment as compared with I/R+GFP (P<0.01, Fig. 5C).

To investigate the anti-apoptotic effect of miR-7a/b in I/R-altered myocardial cells *in vivo,* we performed TUNEL assay of rat heart tissue. After I/R, the rate of cell apoptosis was significantly increased as compared with sham controls (P<0.01, Fig. 5D). miR-7a/b mimic treatment attenuated this increase but inhibitor treatment exacerbated this increase as compared with I/R+GFP (P<0.01, Fig. 5D). Therefore, overexpression of miR-7a/b decreased the cardiac infarct damage and reduced I/R-induced myocardial cell apoptosis after I/R *in vivo* in rats.

## Discussion

Here, we investigated the role of miR-7 during myocardial I/R injury *in vitro* and *in vivo.* miR-7a/b was upregulated in H9c2 cells after 10 h hypoxia and 2 h reoxygenation and directly targeted and inhibited the expression of PARP *in vitro* and *in vivo*. Overexpression of miR-7a/b by miR-7a/b mimic reduced SI/R-induced cell apoptosis *in vitro*, and transfection of a lentivirus with miR-7a/b mimic significantly decreased cell apoptosis and cardiac infarct size in a rat I/R injury model. Therefore, overexpression of miR-7a/b reduced myocardial cell apoptosis in rat I/R injury by negatively regulating its target gene, PARP.

MiRNAs are small, non-coding RNAs that play an important role in the regulation of gene expression [32]. A growing body of evidence shows that miRNAs play a pivotal role in heart diseases [1], [33]–[34]. Some miRNAs are associated with cardiomyocyte I/R injury. For example, miR-1 enhances cardiomyocyte apoptosis by regulating the target genes Hsp60 and Hsp70, whereas miR-133 targets and represses caspase-9 expression to decrease cardiomyocyte apoptosis [35]. miR-320 was downregulated after I/R in murine hearts, and knockdown of miR-320 reduced I/R-induced cardiomyocyte apoptosis by suppressing Hsp20 [13]. miR-499 inhibited cardiomyocyte apoptosis in I/R; the putative targets of miR-499 are α and β isoforms of the calcineurin catalytic subunit, and miR-499 protected cardiomyocyte by suppressing calcineurin-mediated dephosphorylation of dynamin-related protein 1 [36]. An adenovirus expressing miR-21 delivered into rat hearts improved left ventricular remodeling and decreased myocardial cell apoptosis in I/R injury [17]. Under hypoxia, miR-199a inhibited hypoxia-inducible-factor 1α (HIF-1α) expression and reduced cardiomyocyte apoptosis. Knockdown of miR-199a during normoxia upregulated the expression of HIF-1α and Sirtuin-1 and induced hypoxia preconditioning [37]. In our studies, the expression of miR-7a/b was sensitive to SI/R in H9c2 cells: after 10 h hypoxia and 2 h reoxygenation, the expression of miR-7a/b was increased by about 2.50-fold as compared with controls. Therefore, miR-7a/b is an I/R-related miRNA in rat cardiomyocyte.

Furthermore, we investigated the potential role of miR-7a/b in I/R-induced myocardial cell injury. Previous studies have confirmed that miR-7 inhibits cell proliferation and increases cell apoptosis in some cancers [23]–[26]. However, whether miR-7a/b is involved in I/R-induced cardiomyocyte apoptosis was not clear. We found that overexpression of miR-7a/b significantly decreased and inhibition of it increased SI/R-induced cell apoptosis *in vitro*. Thus, miR-7a/b may protect cardiomyocyte against I/R injury at the cellular level. These effects were further confirmed *in vivo* after I/R injury with transfection of a lentivirus with miR-7a/b mimic or inhibitor into the rat myocardium. Overexpression of miR-7a/b decreased and its inhibition increased cardiomyocyte apoptosis, myocardial infarct size, AAR and LDH release. Therefore, overexpression of miR-7a/b may protect rat cardiomyocyte against I/R injury *in vivo* and *in vitro*.

MiRNAs enforce their function via degradation or translational inhibition of their target mRNAs [4]–[7] at the post-transcriptional level [8]–[10]. PARP, as a substrate of caspase-3, is closely related to cell apoptosis. We used an miRNA target prediction program and predicted miR-7a/b binding sites at the 3′ UTR of PARP. Transfection with miR-7a/b mimic significantly reduced the luciferase activity of GV126-PARP-3′-UTR-WT. Furthermoreapp:addword:furthermore, in *in vivo* and *in vitro* experiments, transfection with miR-7a/b mimic significantly inhibited but miR-7a/b inhibitor substantially increased the expression of PARP. Therefore, PARP is a functional target gene of miR-7a/b involved in miR-7a/b–mediated cardiomyocyte protection against I/R.

PARP is a substrate for caspase-3, a key protease during apoptosis [38], and cleaved PARP is an important marker for apoptosis [39]. We found the expression of cleaved PARP remarkably reduced after transfection with miR-7a/b mimic in H9c2 cells during SI/R as compared with SI/R alone. In contrast, inhibition of miR-7a/b increased the expression of cleaved PARP as compared with SI/R alone. The expression pattern for cleaved caspase-3 was similar to that of cleaved PARP. Activated caspase-3 weakens the function of DNA repair [19], causing cells to exhibit typical characteristics of apoptosis, including nuclear changes, chromatin condensation, and DNA fragmentation [40]. Therefore, overexpression of miR-7a/b may be responsible for decreasing cell apoptosis and protecting cardiomyocyte during I/R injury.

### Conclusions

Our findings reveal that miR-7a/b is sensitive to SI/R in cardiomyocyte, and overexpression of miR-7a/b protects cardiomyocyte against I/R-induced apoptosis *in vitro* and *in vivo*. PARP may be the target gene of miR-7a/b against I/R-mediated cardiomyocyte apoptosis. miR-7a/b may be a potential drug target for treating cardiomyocyte I/R injury.
